# CRISPR/Cas12a Technology Combined with Immunochromatographic Strips for the Portable Detection of SFTS Bunyavirus

**DOI:** 10.4014/jmb.2603.03014

**Published:** 2026-06-15

**Authors:** Hao Tang, Yuqing Xing, Guoping Lu, Jilu Shen, Qiang Zhou

**Affiliations:** 1 Department of Clinical Laboratory, the Second Affiliated Hospital of Anhui Medical University, P. R. China; 2 The First Affiliated Hospital of Anhui Medical University, P. R. China; 3 Anhui Public Health Clinical Center, P. R. China; 4 Department of Laboratory Medicine, the Fuyang Affiliated Hospital of Anhui Medical University, P. R. China

**Keywords:** CRISPR-Cas12a, Immunochromatography, Portable detection, Loop-mediated Isothermal amplification, SFTSV

## Abstract

Severe Fever with Thrombocytopenia Syndrome (SFTS), caused by SFTS virus (SFTSV), is a widely distributed infection with significant mortality. Diagnosis in resource-limited settings remains challenging. For rapid and convenient diagnosis, we developed a portable rapid diagnosis method that combines Clustered Regularly Interspaced Short Palindromic Repeats (CRISPR)/Cas12a technology with immunochromatographic test strips. The SFTSV RNA was amplified by reverse transcription loop-mediated isothermal amplification (RT-LAMP). The homologous target sequence and single stranded DNA (ssDNA) reporter gene were cleaved by CRISPR/Cas12a in parallel, and ssDNA probes labeled with FAM fluorescein and biotin were captured by an immunochromatographic strip. Finally, the signal on the immunochromatographic strips became visible to the naked eye. Based on CRISPR/Cas12a, a rapid SFTSV detection method was developed, featuring simplicity, rapidity, low cost, and ease of use. The method was applied for the nucleic acid detection of SFTSV in 40 clinical serum samples and compared with RT-polymerase chain reaction (PCR). The new method showed 100% sensitivity and 100% specificity with a detection agreement rate of 100%. The minimum detection limit of the method was 2.5 copies/μL, and no cross-reactivity with nucleic acids from other common pathogens was observed. Detection can be completed within 80 min, and results are observable with the naked eye. For the analysis of clinical samples, the method exhibits good detection performance and thus provides an attractive option for the nucleic acid detection of SFTSV in point-of-care and resource-limited medical settings.

## Introduction

The Severe Fever with Thrombocytopenia Syndrome Virus (SFTSV) is classified by the World Health Organization as one of the most perilous pathogens, exhibiting a mortality rate of 12-30% and being linked with the characteristic thrombocytopenia syndrome [1, 2]. Progressive viral replication and severe thrombocytopenia are the key features of the SFTSV infection with fatal outcomes [3]. With the increasing incidence of the Severe Fever with Thrombocytopenia Syndrome (SFTS) and the rapid spread of the SFTSV vector worldwide, this virus has pandemic potential and poses an imminent threat to global public health [4]. Therefore, prevention and control of SFTSV is critical.

The main clinical manifestations of SFTS are high fever, thrombocytopenia, leukopenia, and gastrointestinal symptoms. In some cases, patients experience severe and rapid disease progression, potentially leading to mortality due to multiple organ failure, including shock, respiratory failure, and disseminated intravascular coagulation [5]. Various pathogens can cause fever with thrombocytopenia, including viral hemorrhagic fever such as hemorrhagic fever with renal syndrome, Xinjiang hemorrhagic fever, dengue fever, and rickettsia-like diseases such as typhus, human ehrlichiosis, and human granulocytic anaplasmosis. Distinguishing SFTS from other diseases with similar symptoms through clinical examination is challenging; therefore, etiological diagnosis plays a crucial role in its identification, clinical treatment, epidemic monitoring, and prevention. Currently, the etiological diagnosis of SFTS predominantly depends on virus isolation, immunological examination, and nucleic acid detection. However, the experimental period of virus isolation is long, cytopathy is not evident, disease progression of SFTS is fast, and the optimal treatment opportunity is easily missed. Although immunological detection methods are relatively cheap and convenient, their accuracy, sensitivity, and long detection window limit their further application. Polymerase chain reaction (PCR) is a rapid and sensitive method; however, its execution necessitates professional technicians and must be carried out in diagnostic laboratories equipped with complex instruments, rendering it unsuitable for on-site testing. Furthermore, the virus primarily spreads through tick bites, and infections predominantly occur in wooded and hilly terrains characterized by limited medical facilities and considerable distances from local medical diagnostic centers. Consequently, patients frequently succumb to delayed diagnosis. Thus, the development of a point-of-care detection method is imperative.

With the advancements in molecular biology, PCR has been consistently utilized for the rapid detection of various pathogens and genes. However, its application still has certain limitations. PCR requires a temperature cycler with a precise temperature control and usually has strict requirements for sample preparation. To overcome the limitations of complex instruments and high requirements for technicians, molecular biology researchers focus on isothermal amplification independent of thermal cyclers. Loop-mediated isothermal amplification (LAMP) is presently the most extensively employed isothermal amplification technique, boasting numerous advantages and widespread use in various fields of nucleic acid amplification. This method utilizes Bst DNA polymerase with a strand displacement activity, uses 4–6 oligonucleotide primers to identify 6–8 regions on the target DNA, and efficiently amplifies the target DNA or RNA at a constant temperature of 65°C [6]. LAMP employs multiple specific primers to enhance selectivity towards the target sequence, thereby reducing the possibility of non-specific amplification. In addition, the sensitivity of LAMP is not affected by inhibitors in the sample and has high specificity [7]. With these advantages, LAMP provides a valuable platform for point-of-care field testing. In particular, when combined with immunochromatographic techniques, it holds great potential for application in resource-limited settings, serving as a core platform for detecting various pathogenic bacteria or viruses [8, 9].

Relevant studies utilizing LAMP technology to detect SFTSV have pushed the sensitivity and portability of LAMP technology to the extreme. Colorimetry, turbidimetry, indicators, and gel electrophoresis can be employed to confirm the final amplification products. However, this ambiguous product validation process fails to accurately identify LAMP amplification products at the base level, increasing the risk of false-positive results. In this context, the DNA Endonuclease Targeted CRISPR Trans Reporter (DETECTR) platform has captured our keen interest.

**Fig. 1:**
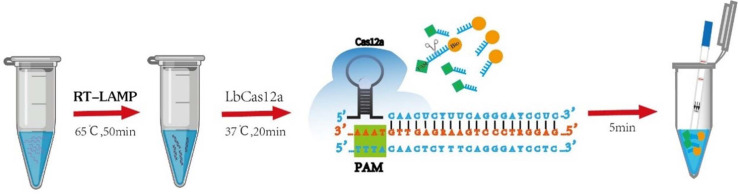
The DETECTR platform.

Nucleic acid detection technology based on Clustered Regularly Interspaced Short Palindromic Repeats (CRISPR) has been developed and has advantages of speed, simplicity, and low cost [10-12]. The Cas12a protein, guided by a guide RNA (gRNA), specifically recognizes a target DNA sequence. The gRNA contains an approximately 20-nucleotide sequence complementary to the target DNA, enabling binding through base pairing. Upon target recognition, the nonspecific trans-cleavage activity of the Cas12 nuclease is activated to cleave the arbitrary single stranded DNA (ssDNA) in the system [13-16]. Through the combination of Cas12a cleavage activity and LAMP, the DETECTR platform was established (Fig. 1) [17]. DETECTR provides high specificity for detection and eliminates false-positive results due to primer mismatches, dimer formation, and cross-contamination during amplification. The DETECTR platform is a versatile tool for detecting various targets, such as West Nile virus, influenza A/B, *Acinetobacter baumannii*, and carbapenemase resistance genes [18-22].

In this study, we established a comprehensive DETECTR detection system for SFTSV. The detection system was used to test serum samples from SFTS patients, and the results were compared with those of RT-PCR to verify its specificity and sensitivity. This work aims to further enhance the application value of LAMP in the rapid detection of SFTSV and to improve the level and efficiency of rapid detection and diagnosis of clinical pathogens.

## Methods

### Reagents, Chemicals, and Machines

All oligonucleotides, PCR primers, LAMP primers, and cloning vectors were synthesized by Shanghai Sangon Biological Co., Ltd.(China). The gRNA and ssDNA probes used in the experiments were provided by Shanghai GeneBiogist techology Co., Ltd.(China). Purified LbaCas12a, Bst 2.0 WarmStart polymerase was purchased from New England Biolabs (USA). Taq DNA polymerase premix and reverse transcriptase were bought from Takara (China). Immunochromatographic lateral flow strips were purchased from Nanjing warbio Biotechnology Co., Ltd. (China). Serum viral RNA extraction kit was obtained from Shanghai Sangon Biological Co., Ltd.

### Virus Samples and RNA Extraction

A total of 40 serum samples were collected from 25 patients with confirmed SFTSV. In accordance with the manufacturer’s instructions, the SFTSV RNA was extracted from 200 μL of serum using the serum viral RNA extraction kit for immediate LAMP and PCR amplification and stored at -80°C for subsequent analysis.

### Design of RT-LAMP and PCR Primers

The SFTSV genome sequences of multiple different strains were retrieved from GenBank (https://www.ncbi.nlm.nih.gov/genbank/), and the SFTSV S fragments of various provinces in China on National Center for Biotechnology Information (NCBI) were compared and analyzed using the SnapGene software. A highly conserved sequence was selected as the amplification region. The GenBank accession numbers of the SFTSV strains used for sequence alignment and primer design are as follows: JQ670933.1. The LAMP primers were designed using PrimerExplorer v.5 (https://primerexplorer.jp/e/) and NCBI-BLAST for template sequence specificity check. The sequences used for LAMP primer design are shown in Supplementary Information 1. Since SFTSV is an RNA virus, multiple site mutations inevitably occur in its genome. Therefore, we added degenerate bases to the primers of LAMP to target more genotypes. Five RT-LAMP primers consisting of two external primers (forward external primers F3 and back external primers B3), two internal primers (forward internal primers FIP and back internal primers BIP), and one ring primer (forward loop primer LoopF) were used for the S fragment (Table 1). In addition, primers from literature [23] were selected for RT-PCR amplification. The sequences of primers are shown in Table 1.

### RT-LAMP and PCR Amplification

**Fig. 2:**
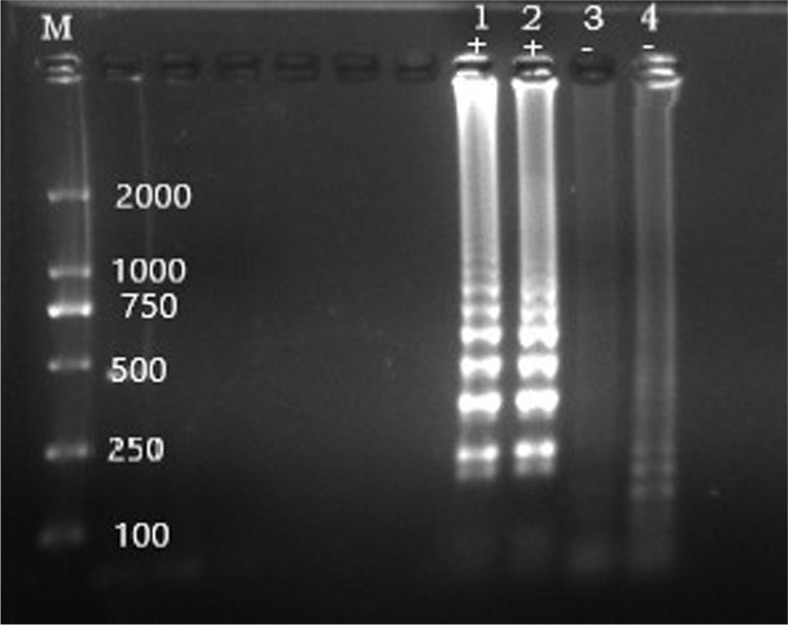
Agarose gel electrophoresis image.

LAMP was conducted according to the manufacturer's instructions. The initial LAMP reaction conditions were adopted from reference [15]. Following optimization of key reaction parameters, the final reaction system was determined as follows: The 25-μL LAMP reaction mixture consisted of 0.5 μL of primer F3 (10 μM), 0.5 μL of primer B3 (10 μM), 1.0 μL of primer BIP (40 μM), 1.0 μL of primer FIP (40 μM), 0.5 μL of primer LF (20 μM), 12.5 μL of 2× reaction mix buffer, 0.5 μL of LAMP fluorescent dye, 4 μL of RNA, and 4.5 μL of ddH2O. The reaction system was incubated at 65°C for 50 min. The LAMP reaction product appeared as a ladder-like band under 2% agarose gel electrophoresis (Fig. 2).

For PCR amplification, the 50-μL reaction mixture consisted of 1 μL of forward primer (20 μM), 1 μL of reverse primer (20 μM), 3 μL of DNA template, and 25 μL of Premix Taq. The remaining volume was supplemented with diethyl pyrocarbonate (DEPC)-treated water. The total reaction system was placed in a PCR machine (ABI Prism 7500, Applied Biosystems, Foster City, CA, USA), and the cycle conditions were set as follows: 94°C for 30 s, 55°C for 30 s, 72°C for 1 min, and the cycle was repeated 30 times. The reaction products were verified by 1% agarose gel electrophoresis.

### CRISPR/Cas12a Detection Reaction

The CRISPR/Cas12a reaction system included the following: 2 μL of cleavage buffer, 2 μL of gRNA (10 μM), 1 μL of Cas12a (1 μM), 3 μL of amplification product, 1 μL reporter (5 μM), and 11 μL of nuclease free water. The reporter was a ssDNA probe labeled with FAM luciferin and biotin at both ends (ssDNA: FAM-TTATTATT-Bio). The reaction mixture was incubated at 37°C for 20 min.

gRNA: UAAUUUCUACUAAGUGUAGAUCAACUCYUUCAGGGAYCCUC.

### Immunochromatographic Lateral Flow Detection

**Fig. 3:**
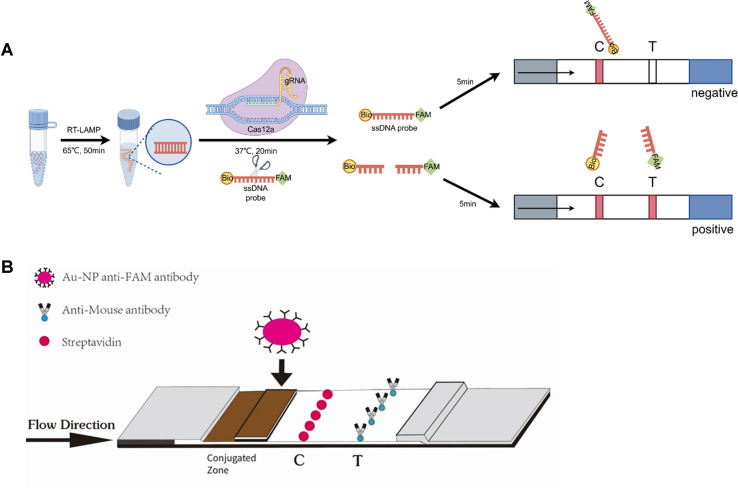
Schematic diagram of the principle of LAMP-Cas12a-LFA (DETECTR). (**A**) Schematic workflow of the DETECTR assay, (**B**) Structure of the immunochromatographic strip

In lateral flow assays, ssDNA probes were labeled with FAM and biotin at the 5′ and 3′ ends, respectively. The CRISPR/Cas12a cleavage reaction product was adjusted to 60 μL volume with water, then inserted with an immunochromatography strip, and incubated at room temperature for 5 min to observe the band change. The absorbent pad of the immunochromatographic lateral flow strip was labeled with a colloidal gold-labeled FAM antibody, the control strip (C) was labeled with streptavidin, and the detection strip (T) was labeled with a goat anti-mouse secondary antibody. The overall detection workflow is illustrated in Fig. 3A, and the detailed structure of the immunochromatographic strip is shown in Fig. 3B.

Negative result: The intact FAM-biotin labeled reporter probe binds to the colloidal gold-labeled anti-FAM antibody. As this complex flows past the control line (C line), it is captured and retained by immobilized streptavidin, resulting in a red color at the C line, while no color develops at the test line (T line).

Positive result: Upon activation, CRISPR-Cas12a cleaves the reporter probe into small fragments, disrupting the linkage between biotin and FAM. Consequently, the gold-labeled anti-FAM antibody cannot be captured at the C line and continues to flow toward the T line, where it is captured by the immobilized secondary antibody, producing a red color at the T line, while the color at the C line is reduced.

### The Limit of Detection (LOD) of the DETECTR

The S fragment of the JQ670933.1 virus strain, published on NCBI, was selected, and the 901–1700 bp sequence was introduced into the pUC57 vector (Shanghai Shengong Biological Co.). The total length of the plasmid is 3510 bp. The plasmid was serially diluted at 1:10 from an initial concentration of 1 ng/μL to serve as a template for LAMP amplification and was detected in sequence until the test line of the immunochromatographic test strip became completely colorless.

### Evaluation of Cross-Reactivity of the DETECTR Assay

To verify the specificity of the proposed method for the target gene and to confirm the absence of cross-reactivity with common viruses, bacteria, and fungi, nucleic acids from Hantavirus, *Orientia tsutsugamushi*, SARS-CoV-2, influenza A virus, Epstein-Barr virus (EBV), *Escherichia coli*, and *Candida albicans* were used as templates in the DETECTR assay.

## Results

### Validation of CRISPR/Cas12a Trans-Cleavage Activity

To directly demonstrate the trans-cleavage activity of Cas12a, we performed a fluorescence-based assay. The FAM-biotin ssDNA reporter used in the LFA was replaced with a FAM-BHQ1 labeled ssDNA probe, while all other reaction components remained unchanged. The reaction mixture was placed in a real-time PCR instrument, and the FAM fluorescence signal was monitored in real time.

**Fig. 4:**
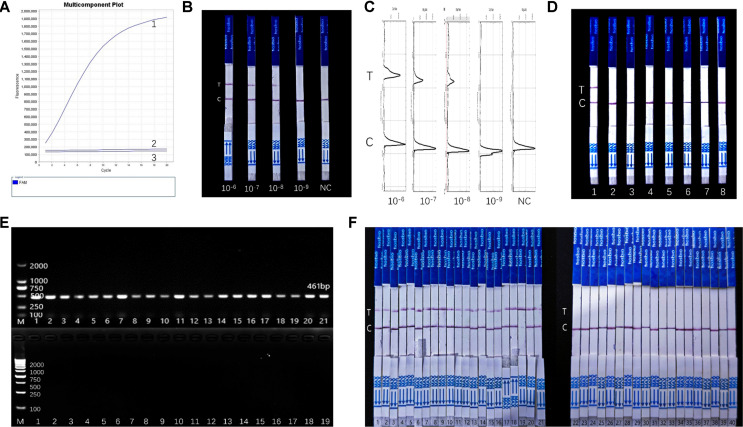
Results of the LAMP-Cas12a-LFA (DETECTR) assay. (**A**) Cas12a trans-cleavage activity. Upon addition of SFTSV LAMP amplicon (Curve 1), FAM fluorescence increased rapidly, indicating probe cleavage. No fluorescence increase was observed without target (Curve 2) or without Cas12a (Curve 3), confirming target-dependent activity. (**B**) Limit of Detection for DETECTR. Serial dilutions of SFTSV plasmid and a negative control were tested. The LOD was determined to be 10^-8^ ng/μL. (**C**) ImageJ analysis of the immunochromatographic strip results. (**D**) Specificity of the DETECTR assay. Nucleic acids from various common pathogens were tested, including: SFTSV (1), Hantavirus (2), *Orientia tsutsugamushi* (3), SARS-CoV-2 (4), influenza A virus (5), Epstein-Barr virus (6), *Escherichia coli* (7), and *Candida albicans* (8). Only SFTSV produced a visible test line (T line). No cross-reactivity was observed with any of the other pathogens tested. C: control line; T: test line. (**E**) RT-PCR results. (**F**) Lateral flow strip results.

As shown in Fig. 4A, upon addition of the SFTSV LAMP amplicon (positive group), a rapid and significant increase in fluorescence intensity was observed, indicating successful cleavage of the probe by activated Cas12a. In contrast, no fluorescence increase was detected in the negative control (without target) or the blank control (without Cas12a).

These results provide direct evidence for the target-dependent trans-cleavage activity of Cas12a and confirm the reliability of our detection mechanism.

### The Limit of Detection of the DETECTR

A positive result was still observed on the lateral flow strip when the plasmid was diluted to 10^-8^ ng/μL (Fig. 4B). ImageJ analysis of the immunochromatographic strip results (Fig. 4C) revealed that the peak intensity of the test line gradually declined with decreasing concentration and became undetectable at 10^-9^ ng/μL. The corresponding T/C intensity ratios are provided in the Supplementary Table S1. Based on these results, the minimum detectable concentration of the DETECTR assay was determined to be 10^-8^ ng/μL. The copy number was calculated using the formula: plasmid copy number (copies/μL) = 6 × 10^23^ × C × 10^-9^ / (N × 660), where C is the DNA concentration (10^-8^ ng/μL) and N is the plasmid size (3510 bp). The LOD of the DETECTR assay was calculated to be 2.5 copies/μL.

### Evaluation of Cross-Reactivity of the DETECTR Assay

As shown in Fig. 4D, except for the positive result of SFTSV, no signal appeared on the test lines for the other pathogens, indicating that the DETECTR assay has no cross-reactivity with several common pathogens.

### Methodological Evaluation of SFTSV Detection by DETECTR

We detected 21 positive and 19 negative clinical samples by RT-PCR (Fig. 4E). In a single-run experiment, the DETECTR assay correctly identified all 21 RT-PCR-positive and 19 RT-PCR-negative clinical samples (Fig. 4F). A comparison of the detection outcomes between the two methods is provided in Table 2.

With RT-PCR as a reference, the sensitivity and specificity of DETECTR were calculated.

To address concerns about sample size and statistical robustness, we calculated the 95% CIs for sensitivity and specificity using the Clopper-Pearson exact method.

Sensitivity = 100% × 21/(21 + 0) = 100% (21/21, 95% CI: 83.9–100%);

Specificity = 100% × 19/(19 +0) = 100% (19/19, 95% CI: 82.2–100%);

Consistency rate of the two methods = 100% × 40/40 = 100%.

A total of 40 clinical samples were tested using real-time quantitative PCR (qPCR) as the reference standard. The qPCR results showed that 21 samples were positive (Ct value < 35) and 19 samples were negative (Ct value ≥ 35), with the positivity cutoff defined as Ct < 35. The DETECTR assay showed 100% agreement with the qPCR results. Detailed results are presented in Supplementary Table S2.

DETECTR demonstrated detection results comparable to those of RT-PCR and qPCR, highlighting its high diagnostic performance and potential for further application and promotion.

## Discussion

With the continuous outbreak of various transmitted diseases and the repeated emergence of new pathogens worldwide, the rapid and effective detection of pathogens has become an urgent demand for the interest of global public health. Hence, the concept of point-of-care testing has emerged.

As a newly discovered virus, the circulation pattern of SFTSV in nature is not fully understood. Ticks are considered to be the main vector of SFTSV. These organisms primarily inhabit hilly regions or forest farms with rich vegetation [24]. Their unique living environment and growth history contribute to the seasonal and regional incidence of SFTS in the nearby population. According to the epidemiological survey data, more than 97% of SFTSV infections occur in farmers working and living in remote villages. However, limited medical resources in these regions delay early diagnosis and treatment of these patients.

LAMP provides an attractive option for SFTSV nucleic acid detection by reducing equipment requirements and speeding up the detection process. This method has significant advantages in sensitivity and portability, but the important aspect of product specificity is not yet discussed and explored. LAMP products are complex, and the conventional fluorescent dye is susceptible to the influence of nonspecific amplification products, resulting in false-positive results. Meanwhile, the most characteristic method, agarose gel electrophoresis, requires long interpretation cycles and slightly cumbersome operation steps. In addition, the huge and bulky UV imager is incompatible with the theme of portable detection. Therefore, high-sensitivity and high-specificity rapid detection can only be achieved when the validation method of LAMP products is also portable.

Recent studies have shown that the CRISPR/Cas12a system has made substantial progress in multiplex detection and complex sample compatibility. Using strategies such as blocker DNA, split-promoter, or active primer production, researchers have successfully achieved highly sensitive isothermal amplification detection of multiple targets—including bacterial 16S rRNA, the Salmonella stn gene, and high-risk HPV 16/ 18—with detection times of 80–90 mins and aM-level sensitivity, validated in serum, drinking water, and clinical samples [25-27]. These strategies are technically complementary to our LAMP-Cas12a-LFA method and have significantly inspired the design and optimization of our study. We hypothesized that if the advantages of LAMP are combined with the characteristics of isothermal cleavage of CRISPR/Cas12a and the cleavage effect is amplified by immunochromatography and colloidal gold, then the specificity of LAMP detection will be significantly improved.

Therefore, based on the CRISPR/Cas12a system, we developed a novel visual detection method for SFTSV and validated its performance using clinical samples. The results demonstrated 100% concordance with PCR detection, a limit of detection of 2.5 copies/μL, and no cross-reactivity with nucleic acids from other pathogens was observed. The above results confirm the excellent diagnostic performance of the proposed method in clinical sample testing.

The high specificity of this method can be attributed to two key factors. First, LAMP employs multiple specific primers that recognize multiple regions of the target sequence, thereby reducing non-specific amplification at the amplification level. Second, the gRNA in the CRISPR/Cas12a system specifically recognizes the target sequence. The trans-cleavage activity of Cas12a is activated only when the gRNA is fully complementary to the target DNA, leading to cleavage of the reporter probe and signal generation. This dual-recognition mechanism (primer recognition plus gRNA recognition) ensures the high specificity of the assay and effectively avoids false-positive results caused by non-specific amplification or primer dimers.

To further assess its practical utility, we systematically compared the key performance characteristics of our LAMP-Cas12a-LFA method with those of other mainstream nucleic acid detection techniques reported in the literature [23, 28-30], including RT-qPCR, conventional RT-LAMP, and RPA-Cas12a-LFA. As shown in Table 3, the LAMP-Cas12a-LFA method strikes a favorable balance among sensitivity, detection speed, cost, and equipment simplicity. These features make it a promising candidate for resource-limited diagnostic settings and support the advancement of SFTS prevention, control, diagnosis, and treatment.

It is worth noting that in previous explorations, we found some variants in the SFTSV genome, which may affect the coverage of the test. To address this, we incorporated degenerate bases into the LAMP primers to achieve broader genotype coverage, although this slightly reduced the efficiency of LAMP amplification. Furthermore, all experiments in this study were performed only once, without technical or biological replicates. Therefore, the intra-assay and inter-assay reproducibility of this method have not yet been formally established. The observed 100% concordance with RT-PCR, while encouraging, represents a single-run observation rather than a fully validated performance metric. In addition, as a qualitative assay, the DETECTR method does not provide quantitative viral load information.

Future multi-center studies with larger sample sizes and rigorous reproducibility protocols are necessary before this method can be considered for clinical application. Meanwhile, both the LAMP reagents and the CRISPR-Cas12 reaction system could be stored as freeze-dried powder, facilitating large-scale production, transportation, and handling. Upon addition of viral nucleic acid, on-site testing could be performed outside clinical diagnostic laboratories—such as at stations, primary emergency departments, and rural clinics—enabling portable detection.

### Ethics Approval and Consent to Participate

This study involved anonymous use of redundant, abandoned patient blood, which is part of the standard treatment agreements with patients in our hospital. This research does not affect patients' health and privacy. The Ethics approval was obtained from the medical ethics committee of Anhui Medical University with the following reference number: LLSC20210802.

## Supplemental Materials

Supplementary data for this paper are available on-line only at http://jmb.or.kr.



## Figures and Tables

**Table 1:** LAMP and PCR primer sequences.

Primer	Sequence (5′→3′)
F3	CATCTGGGCCAAGGATYCC
B3	CCTGATGGAGGCYTACTCYY
FIP	TYCATGCTGCTGTGAACTCTGT-CTTGGCCTTCAGCCACTT
BIP	AACYTCTGTCTTGCTGGCTCCR-GGCARGATGCCTTCACCAA
LoopF	TYCCCAATGATGTTCGGGT
PCR-F	CATCATTGTCTTTGCCCTGA
PCR-R	AGAAGACAGAGTTCACAGCA

Note: Y = C/T, R = A/G

**Table 2:** Comparison of DETECTR and RT-PCR results.

Methods	DETECTR		
RT-PCR	Positive	Negative	Total
Positive	21	0	21
Negative	0	19	19
Total	21	19	40

**Table 3:** Comparison of detection methods for SFTSV.

	LAMP-Cas12a-LFA (DETECTR)	RT-qPCR	RT-LAMP (without CRISPR)	RPA-Cas12a-LFA
Total assay time	60–80 min	90–120 min	40 min	50–80 min
Bulky instrument required	No	Yes	No	No
Specificity	High (6 primers recognize 8 regions; Cas12a activated only upon crRNA-target hybridization)	Moderate	Poor (lack of product verification)	Moderate (2 primers)
Cost per test	Low	Moderate	Moderate	High
Limit of detection (LOD)	2.5 copies/μL	1–100 copies/μL	1–10 copies/μL	1–10 copies/μL
Reagent availability	Low-cost Bst polymerase, widely available, single-enzyme system, stable	Easy	Easy	Expensive RPA enzymes, limited suppliers, multi-enzyme system
Readout method	Lateral flow strip (visual, instrument-free)	Fluorescence curve (instrument-dependent)	Color change/turbidity (visual)	Lateral flow strip (visual, instrument-free)
Training requirement	Minimal	High	Low	Moderate
