# Supplementary material for: CRISPR/Cas12a Technology Combined with Immunochromatographic Strips for the Portable Detection of SFTS Bunyavirus
Source: J Microbiol Biotechnol. 2026 Jun 15;36:e2603014. doi: 10.4014/jmb.2603.03014 (PMC13334080; doi:10.4014/jmb.2603.03014)
Supplement: Supplementary file 1 [file jmb-36-e2603014-supple.pdf]

J. Microbiol. Biotechnol. 2026. 36: e2603014

https://doi.org/10.4014/jmb.2603.03014

1. JQ670933.1. **S segment of SFTSV (901-1300bp)**

GAGGTCTAATTTTGTCGAATTGGATCATGAAATTTAGCCTAATTGGATATGTC
AAATTGCTGCTTACAGGTTCCTGTAAGCAGCAGCAGCAACCTCAGCAGCTC
TGCTGGGGACCCCATCTGGGCCAAGGATTCCCTTGGCCTTCAGCCACTTCA
CCCGAACATCATTGGGGAAGAAGACAGAGTTCACAGCAGCATGGAGAGGA
TCCCTGAAGGAGTTGTAAACTTCTGTCTTGCTGGCTCCGCGCATCTTCACAT
TGATAGTCTTGGTGAAGGCATCTTGCCATAAGGAGTAAGCCTCCATCAGGG
TCTTGGTCGTGGCTTCAGACACCCCTGCAGTTGGAATCAGGGACCCGAAG
GCCATGCACATCATCTCAGGGGGATAATTCTCAACCTTCAGG

2. Supplementary Table S1. The test line/control line (T/C) intensity ratios.

Concentration (copies/μL)	T/C ratio	Result
10^{-6}	0.766	Positive
10^{-7}	0.428	Positive
10^{-8}	0.324	Positive
10^{-9}	0.046	Negative
Negative control	0.023	Negative

Note: T represents the maximum grayscale value of the test line, C represents the maximum grayscale value of the control line, and T/C represents the ratio of the two.

3. Supplementary Table S2. Comparison of qPCR Ct values and DETECTR assay results for 40 clinical samples.

Sample No.	qPCR (Ct value)	qPCR Result	DETECTR Result	Consistency
1	26.760	Positive	Positive	Consistent
2	30.533	Positive	Positive	Consistent
3	29.142	Positive	Positive	Consistent
4	26.8	Positive	Positive	Consistent
5	33.610	Positive	Positive	Consistent
6	29.884	Positive	Positive	Consistent
7	28.511	Positive	Positive	Consistent
8	25.170	Positive	Positive	Consistent
9	20.033	Positive	Positive	Consistent
10	34.788	Positive	Positive	Consistent
11	33.626	Positive	Positive	Consistent
12	25.754	Positive	Positive	Consistent
13	27.306	Positive	Positive	Consistent
14	23.426	Positive	Positive	Consistent
15	24.288	Positive	Positive	Consistent
16	31.447	Positive	Positive	Consistent
17	33.906	Positive	Positive	Consistent
18	27.310	Positive	Positive	Consistent
19	23.081	Positive	Positive	Consistent
20	30.491	Positive	Positive	Consistent
21	33.463	Positive	Positive	Consistent
22	⩾35	Negative	Negative	Consistent
23	⩾35	Negative	Negative	Consistent
24	⩾35	Negative	Negative	Consistent
25	⩾35	Negative	Negative	Consistent
26	⩾35	Negative	Negative	Consistent
27	⩾35	Negative	Negative	Consistent
28	⩾35	Negative	Negative	Consistent
29	⩾35	Negative	Negative	Consistent
30	⩾35	Negative	Negative	Consistent
31	⩾35	Negative	Negative	Consistent
32	⩾35	Negative	Negative	Consistent
33	⩾35	Negative	Negative	Consistent
34	⩾35	Negative	Negative	Consistent
35	⩾35	Negative	Negative	Consistent
36	⩾35	Negative	Negative	Consistent
37	⩾35	Negative	Negative	Consistent
38	⩾35	Negative	Negative	Consistent
39	⩾35	Negative	Negative	Consistent
40	⩾35	Negative	Negative	Consistent

Note: The positivity cutoff for qPCR was set at Ct < 35. Samples with Ct values ≥ 35 or undetermined were considered negative.
